# Initial symptom presentation after high school football-related concussion varies by time point in a season: an initial investigation

**DOI:** 10.1186/s40798-018-0121-8

**Published:** 2018-01-31

**Authors:** Benjamin L. Brett, Andrew W. Kuhn, Aaron M. Yengo-Kahn, Zachary Y. Kerr, Christopher M. Bonfield, Gary S. Solomon, Scott L. Zuckerman

**Affiliations:** 1 Department of Psychology, Veterans Affairs Connecticut Healthcare System, West Haven, CT USA; 2 Department Counseling, Educational Psychology and Research, University of Memphis, Memphis, TN USA; 3 Vanderbilt Sports Concussion Center, Vanderbilt University School of Medicine, Nashville, TN USA; 4 Department of Neurological Surgery, Vanderbilt University Medical Center, Nashville, TN USA; 5 Department of Exercise and Sport Science, University of North Carolina, Chapel Hill, NC USA

**Keywords:** Sport-related concussion, Symptoms, Modifying factors, Neurocognitive function, ImPACT testing, Sport injury

## Abstract

**Background:**

Schedule-based and in-season factors (e.g., competition type) have been shown to be associated with symptom reporting patterns and injury severity in sport-related concussion (SRC). To determine if acute neurocognitive and symptom presentation following SRC differ by time point within a high school football season.

**Methods:**

Multicenter ambispective cohort of high school football players who sustained a SRC (*N* = 2594). Timing (early, mid, and late season) of SRC was based on median dates for the start of the pre-season, regular season, and playoffs of each states’ football schedules. Analysis of covariance (ANCOVA) investigated differences across season period groups for: (1) neurocognitive test scores, (2) total symptom scores (TSS), and (3) individual symptom increases from baseline within 1-week post-injury.

**Results:**

Significant group differences were observed in TSS, *F*(2, 2589) = 15.40, *p* <  0.001, *η*_p_^2^ = 0.01, and individual symptom increases from baseline, *F*(2, 2591) = 16.40, *p* <  0.001, *η*_p_^2^ = 0.01. Significant increases were seen from baseline to both midseason and late season in both TSS, *χ*^2^ = 24.40, *p* <  0.001, *Φ* = 0.10 and individual symptoms, *χ*^2^ = 10.32, *p* = 0.006, *Φ* = 0.10. Post hoc tests indicated a linear trend, with late-season injured athletes reporting approximately twice the TSS (13.10 vs. 6.77) and new symptoms (5.70 vs. 2.68) as those with early-season injuries.

**Conclusion:**

In a cohort of American high school football student-athletes, those suffering SRC in the late-season time period had increased acute symptom burden. SRC sustained later in-season may require more conservative management.

## Key points

- A “seasonality effect” was observed where American high school football players who sustained a SRC later in the season reported significantly increased total symptom scores and number of individual symptoms compared to earlier season SRCs.
- Sports medicine professionals should be aware of external and situational factors affecting symptom reporting following SRC.
- The symptomology and presentation of SRC may vary significantly over the course of the football season.

## Background

Sport-related concussion (SRC) accounts for 25–50% of all concussions sustained by children [1, 2] and occurs in roughly 1.1 to 1.9 million US athletes ≤ 18 years [3]. SRC has become an international health concern, with public attention paid primarily to professional and elite athletes [2, 4]. However, given the large number of youth athletes participating in contact sports every year [5, 6], the overall burden of this public health problem rests at the youth and high school level [7]. American football, in particular, accounts for 41% of all high school SRCs [7]. Two recent studies from the 2008/2009–2012/2013 and 2011/2012–2013/2014 seasons estimate the current rate of SRC among high school football players to be 8.2–9.21/10,000 athletic exposures [8, 9].

Concomitant with an increase in SRC diagnoses is heightened public awareness, healthcare utilization, and research [10–12]. A recent report by the Institute of Medicine called for more research surrounding SRC risk in athletes aged 5 to 21 years [13]. Several efforts have been directed towards the role of biopsychosocial factors influencing SRC incidence, presentation, and recovery. Several modifying factors have been found to influence both SRC incidence [14–17] and prolong recovery [18–24]. In the acute post-concussion period, factors such as sex [25], history of concussion [26, 27], ADHD [28], and age [15, 29] have been shown to increase neurocognitive deficits, while sex or pre-existing psychiatric disorders have been associated with higher acute total symptom scores [30–32].

The identification of other sport-related modifying factors, beyond biopsychosocial variables, may also prove worthwhile. For instance, schedule-based and in-season factors, such as competition type [33–35] and injury mechanism [36], have been shown to be associated with symptom reporting patterns acutely and during recovery.

One trend that has yet to be investigated is the effect of seasonality, defined operationally as time point in the season, on acute presentation (symptom reporting and neurocognitive functioning) after SRC. The primary objective of this study was to investigate the effect of seasonality (early, mid, and late season) on acute neurocognitive performance and symptom burden following SRC in American high school football players. Due to the exploratory nature of this study, we adopted the null hypothesis, proposing that there would be no differences in initial presentation following SRC across season period groups.

## Methods

### Study design and overview

A retrospective analysis of prospectively collected data (ambispective design) was conducted. Participants included 2674 student-athletes from various high schools across the USA who underwent routine pre-season and post-concussion neurocognitive testing using Immediate Post-Concussion Assessment and Cognitive Testing (ImPACT) [37]. Anonymous, deidentified data were obtained for the study from the lead programmer at ImPACT, who was blinded to the purpose of the study. Due to the deidentified nature of the data abstraction, repeat injuries in the same athlete could not be controlled. Institutional Review Board (IRB) approval was obtained prior to analysis (IRB# 120991), and the study was performed in accordance with the standards of ethics outlined in the Declaration of Helsinki.

### Selection of participants

Following written, informed consent by the student-athlete and/or his parent/guardian, all participants completed a baseline neurocognitive test as part of routine athletic care. Baseline ImPACT testing was conducted in group settings during the pre-season and under the supervision of a sports medicine professional trained in the administration of ImPACT [38]; however, group sizes or administration procedures may have slightly varied across sites. High school football players who sustained a SRC from 2011 to 2016 were included in the analysis. Football was chosen due to its highest concussion rate among high school athletics [39]. Additionally, football was the only sport investigated to standardize season time points and to minimize potential confounds, such as different season durations and game schedules.

SRC diagnoses were made in accordance with the definition provided by the 2008 and 2012 International Concussion in Sport Group (CISG) guidelines [40, 41]. Diagnoses were made by certified athletic trainers (ATCs) or team physicians based on the following on-field/sideline signs or symptoms: (1) lethargy, fogginess, headache, dizziness, nausea, visual problems, photophobia, or phonophobia; (2) alteration in mental status; (3) loss of consciousness; or (4) amnesia. Grading systems of concussion severity were not utilized, based on the aforementioned CISG guidelines [40, 41]. All athletes included in the study completed a post-injury ImPACT test within 7 days of injury (M = 4.21, SD = 1.68). Assessments that obtained a positive invalidity indicator, as designated by ImPACT [37], were not considered as eligible for the study and were excluded. Due to the inclusion of only valid tests as part of the data extraction, the precise number of invalid cases could not be specified. Of the total 2674 individual athletes who met these criteria, those with missing data (*n* = 48, 1.8%) and reporting English as a second language (*n* = 32, 1.2%) were excluded, resulting in a final sample of 2594 student-athletes.

### Data collection

All data were obtained from ImPACT, including basic demographic and biopsychosocial information, four indices of neurocognitive functioning, and a self-reported symptom inventory [37]. The four individual neurocognitive indices yield composite scores for verbal memory, visual memory, visual motor speed, and reaction time. The self-report symptom inventory computes a total symptom scale (TSS), comprised of 22 common symptoms, each rated on a 0-6 Likert scale, with 0 = none and 6 = severe.

### Seasonality

Season period ranges (early, mid, and late season) were defined based on published data/reports from all 50 states (+District of Columbia) for dates of high school football pre-season, regular season, playoffs, and final game. Following the precedent set by previous studies investigating injury rate differences across season periods, cut-points for group formation included pre-season (early), regular season (mid), and postseason (late) [42, 43]. Median dates for the start of pre-season, regular season, and playoffs across all available states were used to determine cut points for ranges. *Pre-season* was defined as training camp, exhibition games, or scrimmages. The median pre-season start date was August 7 (range = July 30 to August 18; data available from 33 states), which was designated as the commencement of the early-season period. *Regular season* was defined as the scheduled games for all teams. The median regular season start date was August 27 (range = August 18 to September 10; data available in 50 states), which was designated as the mid-season cutoff date. *Late season* was defined as the playoff games, where teams played single elimination games, based on record and seeding. The median playoff start date was November 5 (October 14–November 29; data was available for 50 states), which was designated as the cutoff of the late-season period. Median data for end of season date was November 26. Athletes were classified into season period groups (early, mid, and late) based on their date of SRC injury, which was extracted from post-injury ImPACT tests. Though these times mirrored pre-season, regular season, and playoffs, some overlap of periods likely existed between regions. Thus, early, mid, and late season were chosen to better represent the amalgamation of schedules.

### Neurocognitive and symptom outcomes

Three a priori outcomes were determined: (1) neurocognitive test scores, (2) total symptom scores (TSS), and (3) increase in individual symptoms. A meaningful change for each outcome measure was defined as follows. For the outcomes of neurocognitive scores and TSS, a reliable change was based on meaningful change at the 80% confidence interval level [44]. To determine increase in number and severity of symptoms, a previously validated cutoff score (2+ symptoms, increased 1+ point) was used to classify athletes as meaningfully changed from baseline [45]. The term “symptom burden” was collectively used when referring to both symptom outcomes (TSS and individual symptom increase).

### Statistical analysis

Demographic and biopsychosocial data from each athlete’s ImPACT evaluation were extracted and compared across seasonality groups using analysis of variance (ANOVA) tests. Variables that significantly differed between the early-, mid-, and late-season SRC groups were selected as covariates for subsequent analysis of covariance (ANCOVA) tests comparing outcome variables (i.e., post-injury scores and symptoms; Table 1). Baseline neurocognitive composite and symptom scores were also entered as covariates for their respective post-injury ANCOVA in order to control for individual differences at baseline. Separate ANCOVA tests were conducted for each post-injury ImPACT composite score and TSS. Due to the recent emphasis on individualized symptoms over TSS [46], differences in the number of symptoms that increased from baseline assessments to post-injury were compared across seasonality groups. Additionally, ANOVA tests were performed to compare individual symptom reporting for each of the 22 symptoms within the PCSS across the three groups, with a Bonferroni-corrected significance (alpha) level set at 0.002 [47]. Separate chi-squared tests were performed to compare rates of reliable change from baseline for neurocognitive and symptom scores. Reliable change was based on meaningful change at the 80% confidence interval level [44]. Additionally, a chi-squared test was performed as a means to test individual symptom increases from baseline based on a previously validated cutoff score (2+ symptoms, increased 1+ point) [45]. Bonferroni-corrected alpha level for multiple comparisons was set at 0.008 [47].

**Table 1:** Baseline characteristics and comparisons of 2594 high school football athletes who sustained concussion in the early, mid, and late season

Mean (SD) or *n* (%)	Early season (*n* = 418)	Midseason (*n* = 2078)	Late season (*n* = 98)	Total (*n* = 2594)	*F*/*χ*^2^	*p* value*
Demographic factors						
Age	15.25 (1.19)	15.31 (1.23)	15.76 (1.23)	15.3 (1.23)	6.97	*0.001*
History of concussion	116 (27.8)	596 (28.7)	34 (35.4)	746 (28.8)	2.29	0.32
Years playing at HS level	1.65 (1.90)	1.73 (1.96)	2.13 (2.40)	1.73 (1.97)	3.88	0.21
Gender (male)	418 (100)	2074 (99.90)	98 (100)	2590	1.00	0.61
Handedness (right)	350 (83.37)	1792 (86.23)	84 (85.71)	2226 (85.81)	2.81	0.59
Country (USA)	418 (100)	2068 (99.50)	98 (100.00)	2584 (99.61)	2.49	0.65
ADHD	58 (13.86)	282 (13.57)	12 (12.24)	352 (13.57)	0.20	0.91
Learning disorder	36 (8.6)	196 (9.43)	10 (10.20)	242 (9.32)	0.33	0.85
Days since injury	4.09 (1.69)	4.24 (1.68)	4.18 (1.73)	4.21 (1.68)	1.52	0.22
Treatment history						
Headaches	44 (10.50)	260 (12.90)	12 (12.80)	316 (12.18)	1.08	0.58
Migraines	36 (9.10)	146 (7.30)	6 (6.40)	188 (7.24)	1.72	0.42
Epilepsy/seizures	0 (0.00)	18 (0.90)	0 (0.00)	18 (0.69)	4.50	0.11
Brain surgery	0 (0.00)	4 (0.20)	0 (0.00)	4 (0.15)	0.99	0.61
Meningitis	2 (0.50)	14 (0.70)	2 (2.10)	18 (0.69)	2.81	0.25
Alcohol/substance abuse	0 (0.00)	2 (0.10)	0 (0.0)	2 (0.07)	0.49	0.78
Depression/anxiety	8 (2.00)	46 (2.30)	2 (2.10)	56 (2.15)	0.15	0.93
Baseline neurocognitive and symptom scores						
Verbal memory	81.73 (10.31)	82.78 (10.16)	83.08 (10.49)	82.62 (10.20)	6.69	*0.001*
Visual memory	72.46 (12.95)	74.14 (12.83)	70.24 (13.90)	73.74 (12.83)	0.82	*0.44*
Visual motor speed	34.72 (6.26)	35.19 (6.95)	35.00 (6.14)	35.11 (6.79)	1.05	*0.35*
Reaction time	0.63 (0.09)	0.63 (0.09)	0.62 (0.08)	0.63 (0.09)	11.02	*< 0.001*
Total symptom score	6.02 (10.60)	4.35 (7.95)	4.54 (8.37)	4.54 (8.37)	1.88	*0.15*

Italics indicates selection of covariates to control for in outcome measure statistical modeling

*p values of one-way ANOVAs (continuous variables) and chi-square analyses (*χ*^2^; binary and categorical variables) for comparison of those who sustained concussion in the early, mid, or late season

## Results

A total of 2594 athletes were included in the final analysis for the early- (*n* = 418), mid- (*n* = 2078), and late-season groups (*n* = 98). Demographic, medical, and neuropsychiatric history, and baseline neurocognitive and symptom scores are summarized (Table 1), along with accompanying between-group comparisons. Timing from SRC to post-injury assessment, measured in days, did not differ significantly between groups: early (M = 4.09, SD = 1.69), mid (M = 4.24, SD = 1.68), and late (M = 4.18, SD = 1.68), *F*(2, 2591) = 1.52, *p* = 0.22. Across all three groups, 458 (17.8%) athletes were evaluated within the first day post injury, 1258 (49.0%) were evaluated at approximate 3 days following injury, and 850 (33.1%) were evaluated between 4 and 7 days. Age emerged as the only factor that significantly differed across groups, *F*(2, 2591) = 6.97, *p* = 0.001, and was entered into ANCOVA tests as a result. Verbal memory, *F*(2, 2591) = 6.69, *p* = 0.001, and reaction time, *F*(2, 2591) = 11.02, *p* <  0.001, significantly differed across groups as well. Regardless of statistical significance, each baseline test was entered as a covariate for their respective post-injury neurocognitive or symptom score as part of each ANCOVA.

### Neurocognitive performance

No significant differences between the three groups were observed for any of the four neurocognitive composite scores (Tables 2 and 3). Classification rates of neurocognitive scores as injured from baseline at the 80% confidence interval did not significantly differ between the three groups (Table 4).

**Table 2:** Comparison of post-injury outcome measures among athletes who sustained an early, mid, and late season concussion

Post-concussion outcome: mean (SD)	Early season (*n* = 418)	Midseason (*n* = 2078)	Late season (*n* = 98)	*F*	*p* value	*η*_p_^2c^
Verbal memory^a^	83.13 (12.99)	83.35 (13.22)	81.76 (14.98)	1.55	0.21	–
Visual memory^a^	73.27 (12.96)	74.60 (14.63)	69.65 (15.18)	2.68	0.07	–
Visual motor speed^a^	35.86 (6.99)	36.21 (7.60)	35.51 (6.80)	0.55	0.58	–
Reaction time^a^	0.63 (0.12)	0.63 (0.12)	0.65 (0.13)	2.00	0.14	–
Total symptom score^a^	6.77 (9.99)	9.31 (14.85)	13.10 (16.80)	15.40	*< 0.001*	0.01
Number of symptoms increased^b^	2.68 (3.59)	3.61 (4.73)	5.70 (5.84)	16.40	*< 0.001*	0.01

Italics indicates significant *p* value based on Bonferroni-corrected alpha levels = 0.008

^a^Each comparison of outcome was controlled for age and respective baseline score in ANCOVA modeling; number of symptoms increased was from baseline report and therefore baseline number of symptoms was not included as a covariate

^b^Increase in number and severity of symptoms based on a previously validated cutoff score (2+ symptoms, increased 1+ point) to classify athletes as meaningfully changed from baseline [45]

^c^Partial *η*^2^ interpretation for effect size; small = 0.01, medium = 0.06, large = 0.14

**Table 3:** Comparison of the number of significant reliable change rates for each outcome measure among athletes who sustained concussion in the early, mid, and late season

Post-concussion outcome *n* (%)	Early season	Midseason	Late season	*χ* ^2c^	*p* value	*Φ* ^d^
Verbal memory^a^	62 (14.83)	352 (16.94)	26 (26.53)	7.72	0.02	–
Visual memory^a^	42 (10.04)	280 (13.47)	16 (16.32)	4.58	0.10	–
Visual motor speed^a^	30 (7.18)	224 (10.78)	16 (16.32)	8.67	0.01	–
Reaction time^a^	78 (18.66)	440 (21.17)	28 (28.57)	4.79	0.09	–
Total symptom score^a^	64 (15.31)	482 (23.20)	36 (36.73)	24.40	*< 0.001*	0.1
Number of symptoms increased^b^	190 (45.45)	1040 (50.05)	62 (63.27)	10.32	*0.006*	0.06

Italics indicates significant *p* value based on Bonferroni-corrected alpha levels = 0.008

^a^Percentages of athletes classified as concussed based on Reliable change indices at the 80% confidence interval level [44]

^b^Increase in number and severity of symptoms based on a previously validated cutoff score (2+ symptoms, increased 1+ point) to classify athletes as meaningfully changed from baseline [45]

^c^*χ* ^2^ analyses comparing the proportion of athletes classified as concussed based on Reliable change indices at the 80% confidence interval level [44]

^d^Phi effect size interpretation values: [df = 2] small = 0.07, medium = 0.21; large = 0.35

**Table 4:** Comparison of individual symptoms for athletes who sustained concussion in the early, mid, and late season

	Descriptive data					Comparison between season time^b^		
	Early	Mid	Late	*F*	*p* value^a^	Early to mid	Mid to late	Early to late
Headache	182 (43.54)	898 (43.21)	58 (59.18)	4.87	0.01	–	–	–
Nausea	12 (2.87)	48 (2.31)	2 (2.04)	0.26	0.77	–	–	–
Vomiting	40 (9.57)	332 (16.00)	16 (16.33)	5.71	0.003	–	–	–
*Balance*	62 (14.83)	426 (20.50)	38 (38.78)	14.38	*< 0.001*	*0.02*	*< 0.001*	*< 0.001*
*Dizziness*	86 (20.57)	490 (23.58)	40 (40.82)	9.12	*< 0.001*	0.38	*< 0.001*	*< 0.001*
Fatigue	40 (9.57)	276 (13.28)	14 (14.29)	2.27	0.10	–	–	–
*Falling asleep*	58 (13.88)	400 (19.25)	32 (32.65)	9.63	*< 0.001*	*0.03*	*0.003*	*< 0.001*
Sleeping more	34 (8.14)	190 (9.14)	18 (18.37)	5.14	0.06	–	–	–
Sleeping less	30 (7.17)	190 (9.14)	14 (14.29)	2.54	0.08	–	–	–
*Drowsiness*	86 (20.57)	566 (27.23)	36 (36.73)	6.72	*0.001*	0.13	0.09	*0.003*
Light sensitivity	76 (18.18)	484 (23.29)	30 (30.61)	4.39	0.01	–	–	–
*Noise sensitivity*	70 (16.75)	480 (23.10)	32 (32.65)	7.12	*0.001*	*0.01*	0.07	*0.002*
Irritability	46 (11.00)	264 (12.70)	16 (16.33)	1.11	0.33	–	–	–
Sadness	30 (7.18)	178 (8.57)	14 (14.29)	2.57	0.08	–	–	–
Nervousness	8 (1.91)	104 (5.00)	8 (8.16)	5.23	0.01	–	–	–
More emotional	18 (4.31)	150 (7.21)	10 (10.20)	3.20	0.04	–	–	–
Numbness	22 (5.26)	82 (3.95)	6 (6.12)	1.19	0.31	–	–	–
*Slowed down*	52 (12.44)	448 (21.56)	38 (38.78)	19.14	*< 0.001*	*< 0.001*	*< 0.001*	*< 0.001*
*Mentally foggy*	40 (9.57)	384 (18.48)	36 (36.73)	22.43	*< 0.001*	*< 0.001*	*< 0.001*	*< 0.001*
*Concentration*	66 (15.79)	572 (27.53)	36 (36.73)	15.69	*< 0.001*	*< 0.001*	0.10	**<** *0.001*
*Memory problems*	28 (6.70)	360 (17.32)	24 (24.49)	17.75	*< 0.001*	*< 0.001*	0.14	*< 0.001*
Vision problems	38 (9.09)	246 (11.84)	20 (20.40)	5.00	0.01	–	–	–

^a^Indicates significant *p* value based on Bonferroni-corrected alpha levels = 0.002

^b^Indicates significant *p* value based on Tukey HSD alpha levels = 0.05

### Total symptom score

**Fig. 1:**
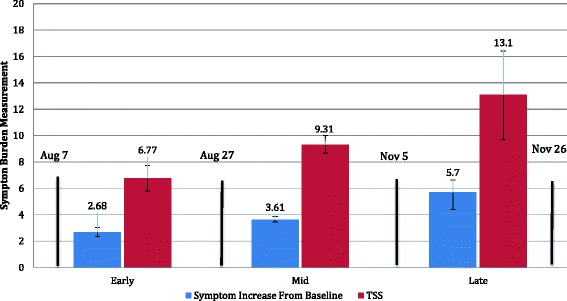
Symptom burden at early-, mid-, and late-season intervals. Early season interval = August 7–August 26; mid-season = August 27–November 4; late season = November 5–November 26. Blue bars indicate increase in number of individual symptoms from baseline. Red bars indicate ImPACT total symptom score (TSS). Error bars reflect 95% CI of the mean

Results revealed significant differences in TSS across all three groups, *F*(2, 2589) = 15.40, *p* <  0.001, and partial *η*_p_^2^ = 0.01 (Tables 2 and 3; Fig. 1). Tukey’s honest significant difference (HSD) post hoc tests indicated that these differences existed at all three levels and were linear, with mid-season athletes reporting significantly higher TSS than early-season athletes (mean difference = 2.54, *p* = 0.001) and late-season athletes reporting significantly higher TSS than mid-season athletes (mean difference 3.75, *p* = 0.01). Late-season athletes reported significantly higher TSS than early-season athletes (mean difference = 6.30, *p* <  0.001). On average, TSS from athletes who were injured in the late-season group were twice that of athletes who were injured early in the season (13.10 vs. 6.77). Significant differences in being classified as reliably changed in TSS from baseline at the 80% confidence interval were observed between the early (15.31%), mid (23.20%), and late season (36.73%) injured athletes, *χ*^2^ = 24.40, *p* <  0.001, and *Φ* = 0.10.

### Individual symptoms

**Fig. 2:**
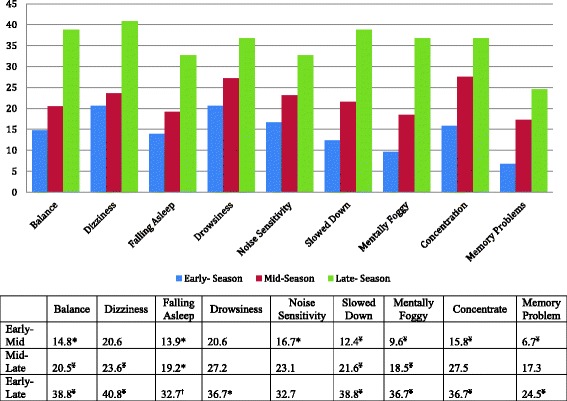
Percentage of athletes reporting symptoms that statistically differed across early-, mid-, and late-season injuries. Asterisk indicates significant at the < 0.05 level. Dagger indicates significant at the 0.01 level. Yen sign indicates significant at the < 0.001 level

A similar trend was observed for the number and severity of increased individual symptoms from baseline, with statistically significant differences across all three groups, *F*(2, 2591) = 16.40, *p* <  0.001, and *η*_p_^2^ = 0.01. The mean increase of individual symptoms above baseline rose in a linear fashion, from early (2.68), mid (3.61), and late season (5.70). Significant differences were again observed at all three levels, with mid-season athletes reporting a greater increase in symptoms from baseline than early-season athletes (mean difference = 0.95, *p* <  0.001) and late-season athletes reporting a greater increase in symptoms from baseline than mid-season athletes following SRC (mean difference = 1.85, *p* <  0.001). Late-season athletes reported significantly more symptoms above baseline than early-season athletes as well (mean difference = 2.80, *p* <  0.001). Similar to TSS, athletes who sustained a SRC during late season reported nearly twice the number of increased symptoms from baseline as athletes in the early-season period (5.70 vs. 2.68). Of the 22 individual symptoms, 9 emerged as statistically different across all three groups (Table 4; Fig. 2). Tukey HSD revealed variation in differences between early-to-mid and mid-to-late, with late-season athletes demonstrating significantly greater symptoms than early athletes for all 9 symptoms. Similarly, significant differences in being classified as injured from baseline based on increases in individual symptoms (2+, 1+ cutoff) were observed between early- (45.45%), mid- (50.05%), and late-season (63.27%) injured athletes, *χ*^2^ = 10.32, *p* = 0.006, *Φ* = 0.10.

## Discussion

The purpose of the current study was to assess the effect of seasonality on acute SRC presentation in a large cohort of American high school football student-athletes within 1-week post-injury. Acute symptom burden, measured by TSS and individual symptom increases, significantly increased as the high school football season advanced. Progressively higher measures of symptom burden were observed across season periods, with athletes in the late-season period reporting twice the post-injury TSS compared to athletes in the early-season period. Similarly, compared to the early-season group, athletes injured in the late-season period reported almost twice as many individual symptom increases from baseline following SRC.

While recovery was not included as an outcome in the current study, initial symptom burden has been identified as one of the strongest and most consistent predictors of prolonged recovery [24, 30, 48–52]. Therefore, more conservative management of athletes who sustain a SRC later in the season may be warranted. Silverberg et al. [53] showed that athletes with higher symptom burden immediately following injury were at increased risk for “symptom spikes” during return to regular activities (RTRA), such as school. Therefore, the timing of injury during the season may influence return-to-school decisions. Clinicians can counsel parents and athletes on the prospect of extended duration for return-to-school and/or return-to-play as a result of late-season injuries, which could reduce concern over lingering symptoms or “symptom spikes” during RTRA.

The effect of season period on acute symptom burden should also be considered in the context of other modifying factors, such as age. Sport participation at the youth and adolescent level places children at higher risk for SRC [20, 54] and prolonged recovery [22, 55]. Should future studies validate the current results, there exist additional implications for those at increased risk of concussion and prolonged recovery, especially youth and adolescent athletes [33, 55]. While the reporting of acute fatigue did not significantly differ across the three groups, the effect of accumulated fatigue may be a factor worthy of consideration for elite adolescent and high school athletes who play their sport year-round. This is especially true when considering a recent meta-analysis demonstrating that higher athletic training loads over time were associated with increased rates of both injury and fatigue [56]. Alongside heightened awareness by healthcare providers regarding how time of injury may be associated with concussion symptomatology, consideration should be given to the number of pre-season games and practice schedules as well.

The reason for increased symptom burden later in the season is not entirely clear and could be due to a myriad of reasons. An explanation of the current findings may be due to athletes’ willingness to report an injury that may preclude a rapid return to play. Athletes may be less likely to report injuries later in the season due to increased desire to play in higher stakes games, which could result in only the more severe injuries being reported. This possibility is further supported given that motivation to not be withheld from competition has been significantly associated with underreporting of concussion [57]. Cumulative head impact burden from the season might also cause a higher manifestation of concussion symptoms, which may lead to increased symptom burden at season’s end [58]. These increased symptoms could also be influenced by other factors coinciding with season progression, such as increased academic demands and stress later in the school year, as compared to the pre-season. Other hypothetical explanations for the current findings are increased intensity of playoff games, higher level of competition with faster and stronger athletes, and suboptimal, colder weather conditions in some areas.

The current study is not without limitation. Our sample of high school football athletes who underwent neurocognitive testing may not be generalizable to athletes from other football settings, sports, or sporting levels. Due to the de-identified process of case selection, it cannot be guaranteed that a post-injury assessment was an athlete’s first concussion in a season. Given that a history of multiple concussions has been associated with increased symptom reporting post-injury, not being able to ensure that a post-injury assessment was an athlete’s first SRC in the season presents as a potential confound [59]. The fact that SRC history did not significantly differ across the three groups suggested that sample selection likely buffered the effect of this possible confound. This is also true of the random selection of data and robust sample size of the study [60]. Further, while cut points for season periods were modeled after two previous studies examining effects of season period, due to state differences, potential for injury time misclassification exists. Regardless, a linear trend and variations around the season period thresholds would not have an effect on the progressive increases in post-injury symptom reporting. Lastly, our findings would also be advanced further if the exact mechanism of increased symptom reporting through the season was identified. This could be accomplished by incorporating measures of fatigue or cumulative head impacts in the examination of symptom reporting trends in different season periods.

## Conclusions

Symptom burden following SRC progressively increases through the advancement of the season in high school American football. While further validation is required, these findings suggest that SRC sustained later in-season may require more conservative management with regard to return-to-learn and play activities. Further study is needed to determine the etiology of greater symptom burden reported later in the season.
